# Microbiome R&D and Business Collaboration Forum, April 11–12, 2016, London, UK

**DOI:** 10.1016/j.ebiom.2016.04.035

**Published:** 2016-04-27

**Authors:** Duc H. Le

## 1 Gut Microbiome and Inflammatory Bowel Disease

Bidirectional brain-gut interactions are highly relevant in human intestinal diseases such as irritable bowel syndrome (IBS), and preclinical studies help shed light on its complexity. Dysbiosis of the bacterial gut microbiome became apparent and microbiome modulation is now considered an important target for treatment. To study the pathology of IBS, Wouter J. de Jonge (Amsterdam, The Netherlands) and colleagues employed animal models for stressful early life events that are known to predispose to IBS at adult age. Transplantation of microbiota from a visceral hypersensitive rat to a normosensitive one transferred the phenotype, suggesting that the microbiome is critical in the stress-induced hypersensitivity. Furthermore, the authors addressed potential mechanisms by which neural signals can affect host defense and microbiome composition in the gut lumen. T lymphocytes were found to be very responsive to neuronal signals; and genetic knockout studies have revealed a critical function of these T cells in regulating anti-microbial peptides by intestinal epithelia. As such, the effect of stress and neuronal activity could relay to host defense mechanisms of the gut epithelia and microbial richness and diversity. Future studies are warranted in the field of microbiome adaptation for IBS pathology.

## 2 Modulation of Gut Microbiota in Obesity and Cardiometabolic Disorders

Changes in gut microbiota are associated with metabolic disorders such as obesity, type 2 diabetes and cardiovascular risk factors. Patrice D. Cani (Brussels, Belgium), Willem M. de Vos (Wageningen, The Netherlands) and collaborators have identified *Akkermansia muciniphila* as a potential target to treat cardiometabolic disorders and inflammation. *A. muciniphila* is a mucin-degrading bacterium living in the gut's mucus layer. The authors demonstrated that feeding mice with *A. muciniphila* reduced bodyweight, fat mass and inflammation, and restored gut barrier function by acting on mucus layer thickness and restoring the production of antimicrobial proteins. The authors also showed that in obese people, the abundance of *A. muciniphila* was inversely related to fasting plasma glucose levels, visceral fat accumulation and adipocyte diameter in subcutaneous adipose tissue. Upon caloric restriction, obese individuals with higher baseline *A. muciniphila* showed improved insulin sensitivity markers and other cardiometabolic risk factors. In summary, strategies to modulate *A. muciniphila* composition in the human gut to treat obesity and cardiometabolic disorders warrant further investigation.

## 3 Liver Diseases and Gut Microbiota

The hepatic portal vein conducts blood from the gastrointestinal tract to the liver, carrying metabolites produced by the gut microbiota making the liver one of the main organ that can be influenced by microbiome composition and activities. Philippe Gérard (Jouy-en-Josas, France) and collaborators demonstrated that a specific dysbiosis in intestinal microbiota (IM) was associated with alcoholic liver disease (ALD) severity in patients. The researchers transplanted germ-free and conventional mice with human IM from alcoholic patients with or without alcoholic hepatitis (AH). Mice receiving IM from an AH patient developed more severe liver inflammation with an increased number of liver T and NK lymphocyte subsets, higher liver necrosis, greater intestinal permeability and higher translocation of bacteria than mice harboring the IM from an alcoholic patient without AH (noAH). Distinct differences in IM composition could be observed, with key deleterious bacterial species being associated with AH and the *Faecalibacterium* genus being associated with noAH. A subsequent transfer of IM from a noAH patient could improve alcohol-induced liver lesions in conventional mice previously transplanted with IM from an AH patient. In conclusion, it may be possible to prevent and manage ALD by IM manipulation.

## 4 Gut Microbiota in Cystic Fibrosis

Fiona Fouhy (Fermoy, Ireland) and colleagues in the CFMATTERS project presented novel longitudinal data on the cystic fibrosis (CF) gut microbiota. The researchers examined the gut microbiota of individuals with CF at stability, during pulmonary exacerbation and post exacerbation, and compared that to non-CF controls. DNA was extracted from fecal samples and the 16S rRNA gene was sequenced on the Illumina MiSeq platform. During exacerbation, changes in microbiota at phylum, family and genus levels were detected before intravenous antibiotic therapy, but the most dramatic changes in microbiota were seen after therapy commencement. Functionality of the gut microbiota was also interrogated using samples from 6 people with CF and 6 controls, with shotgun metagenomic sequencing and metabolomic analysis. Pathways involved in lipid metabolism and xenobiotic degradation increased in the CF group compared to the controls. Metabolites were also altered. This study highlights temporal changes in CF gut microbiota, metabolites and microbiota functionality.

## 5 Skin Microbiota in Health and Disease

While the gut microbiota is intensively investigated, knowledge about the skin microbiota, its protective function and immunomodulatory properties remains limited. The skin microbiota of the face and upper back is dominated by *Staphylococcus* and *Propionibacterium* species; in particular, *Propionibacterium acnes* predominately colonizes sebaceous areas. Using comparative genomics analysis, Holger Brüggemann (Aarhus, Denmark) and colleagues revealed the multi-phyletic composition of *P. acne*; certain *P. acnes* phylotypes are associated with healthy skin while others are associated with skin disorders such as acne vulgaris and progressive macular hypomelanosis. Confocal microscopy revealed the colonization pattern of *P. acnes* within the lumen of sebaceous follicles; healthy skin contained an organized, biofilm-like network of bacteria that does not seem to get in close contact with the adjacent keratinocyte layer. In contrast, in acne-affected skin, *P. acnes* was found to be tightly associated to skin cells, which might lead to the activation of innate immune responses. The data highlighted the fragile balance between *P. acnes* and the skin microenvironment, and a dysbiosis in *P. acnes* phylotype composition may lead to skin disorders.

## 6 Skin and Hair Aging, Lifestyle and the Skin and Scalp Microbiomes

William W. Mohn (Vancouver, Canada) reported on a cross-sectional study investigating the skin and scalp microbiome in relation to skin and hair aging and lifestyle, and involving 495 subjects 10–78 years of age. Diversity of the scalp microbiome increased with age, but its overall composition was not correlated with age. The reverse was true of the forehead microbiome. The authors also showed that specific microbial populations were correlated with visible signs of skin and hair aging and lifestyle factors. Many forehead populations were associated with age and, more weakly, with age-related characteristics like periorbital wrinkling and facial hyperpigmentation, while the reverse was true for scalp populations. Overall, facial skin and scalp have distinct microbiome communities uniquely associated with skin and hair aging and lifestyle factors. Two *Corynebacterium* populations exhibited a striking pattern of co-exclusion on both the forehead and scalp. One population was abundant on most subjects in younger age classes, but it appeared to be completely displaced by the other during middle age.

